# Predicting thrombotic risk in patients with classical Hodgkin lymphoma: Thro‐HL multicenter study

**DOI:** 10.1002/hem3.70163

**Published:** 2025-07-13

**Authors:** Giovanni Manfredi Assanto, Eleonora Alma, Alessandro Cellini, Giovanni Marsili, Gianluca Maiorana, Cristina Santoro, Martina Salvatori, Natalia Cenfra, Vladimir Otasevic, Darko Antic, Gianna Maria D'Elia, Maria Paola Bianchi, Giorgia Annechini, Silvio Ligia, Alessandro Pulsoni, Agostino Tafuri, Andrea Visentin, Alfonso Piciocchi, Stefan Hohaus, Maurizio Martelli, Ilaria Del Giudice, Antonio Chistolini

**Affiliations:** ^1^ Department of Translational and Precision Medicine, Hematology Sapienza University of Rome – AOU Policlinico Umberto I Rome Italy; ^2^ Dipartimento di scienze di laboratorio ed ematologiche, IRCCS Policlinico Gemelli Università Cattolica del Sacro Cuore Rome Italy; ^3^ Department of Medicine DIMED Hematology Unit Padova Italy; ^4^ GIMEMA Foundation Rome Italy; ^5^ Department of Clinical and Molecular Medicine, Hematology Unit University Hospital Sant' Andrea – Sapienza Rome Italy; ^6^ Hematology Unit S. Maria Goretti Hospital, AUSL Latina Latina Italy; ^7^ Clinic for Hematology University Clinical Center of Serbia Belgrade Serbia; ^8^ Parexel International Durham North Carolina USA; ^9^ Faculty of Medicine University of Belgrade Belgrade Serbia; ^10^ Hematology, IRCCS Policlinico Gemelli Università Cattolica del Sacro Cuore Rome Italy

## Abstract

Thrombosis Lymphoma (ThroLy) and Khorana scores have been conceived to predict the thrombotic risk in oncohematologic patients. Currently, there is no univocal indication to perform thromboprophylaxis in classical Hodgkin lymphoma (cHL). We performed a retrospective study to validate scores and risk factors in a cohort of consecutive patients with cHL, treated from 2014 to 2022 outside clinical trials. A total of 470 cHL patients without thromboprophylaxis were included, of whom 57 (12%) experienced a thrombotic event (TE) at 3.3 months (range 1–52) from diagnosis. Neither Khorana nor ThroLy score significantly predicted the thrombotic risk. In a multivariate analysis including Throly parameters and other risk factors, an independent prognostic impact on the TE risk was found for bulky disease (3 points), ECOG PS 2–4 (2 points), presence of peripherally implanted central venous catheter (2 points), mediastinal involvement (1 point), which were combined in a new risk model (Thro‐HL). Low‐risk (score 0–1; 39%, *n* = 183), intermediate‐risk (score 2–3; 46%, *n* = 214), and high‐risk (score > 3; 15%, *n* = 72) patients had a significantly different TE rate, of 2.7%, 16%, and 25% (P < 0.001), respectively. Three‐year‐thrombotic event‐free survival was 97% (CI 95–100) for low‐risk and 76% (CI 66–86) for high‐risk patients (P < 0.0001, Harrel's *C*‐index = 0.70). Thro‐HL could be a promising tool to be validated in larger series.

## Graphical abstract

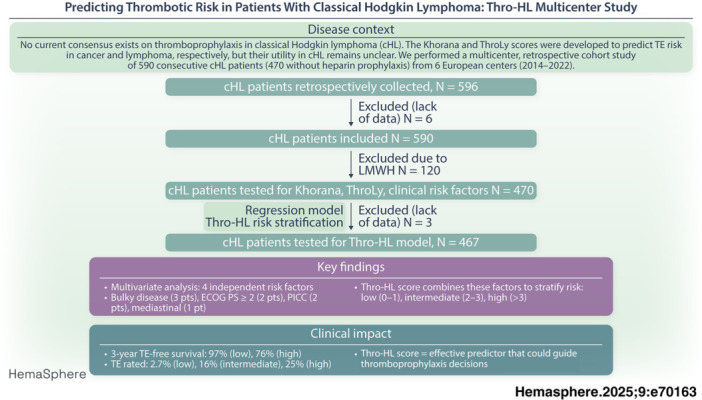

## INTRODUCTION

Cancer patients carry an increased risk of developing thrombotic events (TEs). The occurrence of venous thromboembolism (VTE) is associated with higher mortality and decreased quality of life.^1^, ^2^, ^3^ Thrombotic risk is heterogeneous and can change according to the type of cancer, treatment plan, and an unknown underlying thrombophilia.^4^, ^5^ For this reason, currently, there is no indication to perform thrombosis prophylaxis or thrombophilia screening in patients with cancer, especially when outpatients.^5^, ^6^, ^7^ Patients affected by lymphoma make no exception, with a thrombotic rate ranging from 3% to 15%.^8^, ^9^, ^10^ This rate can vary according to the histological type, being aggressive non‐Hodgkin lymphoma (NHL) at the highest risk with respect to patients with indolent NHL or classical Hodgkin lymphoma (cHL). In the literature, most data are available from patients affected by lymphoma without histological distinction.^9^, ^10^, ^11^, ^12^ Few analyses have been specifically conducted on cohorts, including exclusively cHL patients.^13^ This issue is relevant when considering that cHL is a curable disease, that involves both young and elderly patients, often associated with bulky disease or mediastinal involvement, whose antilymphoma treatment is homogenous for most patients in contrast to thromboprophylaxis strategies. Indeed, there is a lack of consensus on TE prophylaxis measures in lymphomas, and their application in common clinical practice is highly heterogeneous. Khorana^5^ and Thrombosis Lymphoma (ThroLy) scores^14^ were conceived to predict the risk of developing TE in cancer and lymphoma outpatients, respectively, both combining cancer and hematological risk factors. Their validation in independent cohorts led to variable results, mostly due to the inclusion of heterogeneous types of malignancies^15^, ^16^ or lymphomas.^17^, ^18^, ^19^, ^20^, ^21^

The purpose of our study was to evaluate and compare the performance of these two models in the specific setting of cHL and analyze other possible factors influencing the TE risk in this category of patients, which could be used to conceive a disease‐specific model.

## METHODS

We performed a multicenter retrospective study of consecutive patients diagnosed with cHL with age ≥ 17 years between 2014 and 2022 and treated at six hematological centers (Sapienza University, Azienda Ospedaliero‐Universitaria [AOU] Policlinico Umberto I [Rome Sapienza], AOU Sant'Andrea [Rome S. Andrea], Ospedale Santa Maria Goretti [Latina], Policlinico Agostino Gemelli [Rome Cattolica], University of Padova [Padova], and Clinic for Hematology, University Clinical Center of Serbia [Belgrade]). The primary objective was to assess the predictive power of Khorana and ThroLy thrombotic risk scores on TE. The secondary objective was to evaluate other possible risk factors that could increase the predictive power of these models, conceiving a disease‐specific score. This study was approved by the local ethical committee and respects the principles of the Declaration of Helsinki.

All patients' clinical and laboratory data were collected through revision of clinical charts. Demographics, performance status, gender, and clinical history were collected together with clinical hematological features and known conditions associated with the thrombotic risk (i.e., hypertension, chronic obstructive pulmonary disease, diabetes, and hypercholesterolemia) and the thrombotic/cardiovascular history. Comorbidities were considered if requiring specific treatment. Bulky disease was considered as the presence of at least one lymph node with max diameter of >7 cm. Ultrasounds with Doppler and color imaging were used to diagnose VTE only in symptomatic patients, and computed tomography angiography was performed in selected cases to detect pulmonary embolism (PE). Patients who received low‐molecular‐weight heparin (LMWH) prophylaxis from diagnosis were excluded from the thrombotic risk prediction. Peripherally implanted central venous catheter (PICC) insertion and PICC‐related clinically significant events (symptomatic thrombosis requiring treatment) were recorded.

Patients were stratified according to the Khorana model into low (0 points), intermediate (1–2 points), or high (3 or more points), based on type of cancer (1 point for lymphoma), prechemotherapy platelet count ≥ 350 × 10^9^/L (1 point), prechemotherapy hemoglobin level < 100 g/L or the use of red cell growth factors (1 point), prechemotherapy leukocyte count > 11 × 10^9^/L (1 point), and body mass index (BMI) ≥ 35 kg/m^2^ (1 point).^5^ Patients were also stratified according to ThroLy score, into low‐risk (0–1 point), intermediate‐risk (2–3 points), and high‐risk categories (>3 points) based on previous VTE/acute myocardial infarction (AMI)/stroke (2 points), obesity (BMI > 30 kg/m^2^, 2 points), mediastinal involvement (2 points), reduced mobility (ECOG 2–4, 1 point), extranodal localization (1 point), neutrophils < 1 × 10^9^/L during therapy (1 point), and hemoglobin level < 100 g/L (1 point).^14^

The definition of thromboembolic event included all venous and arterial TE, as in Throly score.^14^

### Statistical analysis

Patient's characteristics were summarized by means of frequencies and percentage values for categorical variables, while continuous variables were described with median values and their relative ranges. The chi‐square test or Fisher's exact test was used for categorical variables, whereas the Wilcoxon rank‐sum test was used for continuous variables. All tests were two‐sided with a significance level of 0.05, and confidence intervals were calculated at a 95% level. Univariate logistic regression was used to evaluate potential risk factors that may influence TE, in addition to risk stratification according to Khorana and ThroLy models. All significant factors in the univariate analysis were included in the multivariate analysis and employed, according to odds ratio (OR) values, to propose a disease‐specific risk score. Thrombotic event‐free survival (t‐EFS) was defined as the time from diagnosis to the date of occurrence of TE or the date of the last follow‐up. The probabilities of EFS were estimated following the Kaplan–Meier product limit method, whereas the results of univariate comparisons were performed according to the log‐rank test. Hazard ratio (HR) estimates were produced using the Cox PH regression model. The area under the ROC curve (AUC) statistic and Harrell's concordance index were computed for the multivariate logistic regression model and the Cox PH regression model to assess model performance. Bootstrap was used to calculate the 95% CI of the AUC statistic. All analyses were performed with R version 4.4.1.

## RESULTS

### Patients' characteristics

**Figure 1:**
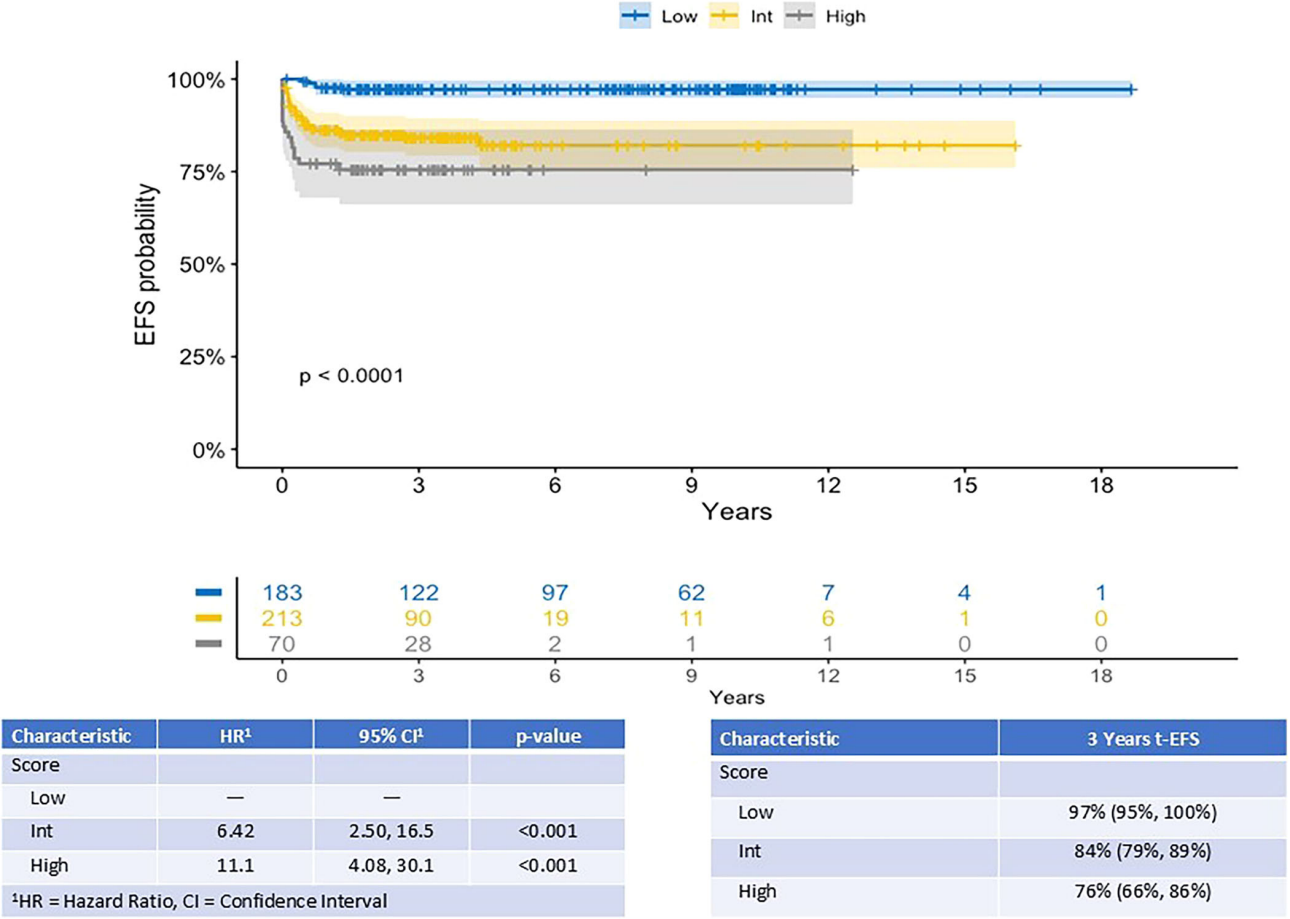
**Thrombotic event‐free survival (t‐EFS) of patients with no primary prophylaxis according to Thro‐HL score.** Event is considered a thrombotic event. Three‐year t‐EFS and HR are reported for each risk group. Concordance (Harrell's *C*‐index): 0.7031646 SD (0.027882).

A total of 596 cHL patients were included in this retrospective study: 132 patients were included from Rome Sapienza, 192 from Rome Cattolica, 140 from Padua, 65 from Rome S. Andrea, 37 from Latina, and 30 from Belgrade. Six were excluded for missing data (Supporting Information S1: Figure 1).

Of 590 patients, 508 (86%) patients received ABVD–AVD‐based first‐line therapy, 53 (9%) BEACOPP‐based treatment, 16 (3%) brentuximab‐vedotin (BV)‐AVD, and 13 (2%) other regimens; 106 (18%) also received consolidation radiotherapy, according to the guidelines at that time (2014–2022). Patients' characteristics are reported in Table 1. Median follow‐up was 3.4 years (interquartile range [IQR] 1.9–6.5).

**Table 1:** Characteristics of patients, according to low‐molecular‐weight heparin (LMWH) primary prophylaxis.

		LMWH prophylaxis		
Characteristic	Overall *N* = 590	No *N* = 470	Yes *N* = 120	P value^a^
Age, median (range)	35 (17, 85)	37 (17, 85)	31 (17, 81)	0.012
Age, *n* (%)				0.020
<60 years	507 (86%)	396 (84%)	111 (93%)	
≥60	83 (14%)	74 (16%)	9 (7.5%)	
Sex, *n* (%)				0.4
Male	291 (49%)	228 (49%)	63 (53%)	
Female	299 (51%)	242 (51%)	57 (48%)	
Ann Arbor stage, *n* (%)				0.14
I–II	306 (52%)	251 (53%)	55 (46%)	
III–IV	284 (48%)	219 (47%)	65 (54%)	
GHSG risk, *n* (%)				<0.001
Early‐stage favorable	93 (17%)	88 (19%)	5 (4.6%)	
Early‐stage unfavorable	169 (30%)	140 (31%)	29 (27%)	
Advanced	299 (53%)	225 (50%)	74 (69%)	
B‐symptoms, *n* (%)	283 (48%)	209 (44%)	74 (62%)	<0.001
Raised LDH, *n* (%)	173 (32%)	126 (29%)	47 (44%)	0.002
Missing	42	28	14	
ESR, median (range)	48 (1, 140)	44 (1, 140)	60 (2, 123)	0.025
Unknown	41	31	10	
Bulky, *n* (%)	139 (24%)	61 (13%)	78 (65%)	<0.001
PICC employed, *n* (%)	296 (50%)	224 (48%)	72 (60%)	0.016
HTA under treatment, *n* (%)	79 (13%)	67 (14%)	12 (10%)	0.2
Diabetes under treatment, *n* (%)	29 (4.9%)	23 (4.9%)	6 (5.0%)	>0.9
R/R disease, *n* (%)	97 (16%)	81 (17%)	16 (13%)	0.3
Anemia, *n* (%)	91 (15%)	73 (16%)	18 (15%)	0.9
Leukocytosis, *n* (%)	245 (42%)	177 (38%)	68 (57%)	<0.001
PLTS > 350 × 10^9^/L, *n* (%)	240 (41%)	175 (37%)	65 (54%)	<0.001
PLTs × 10^9^/L, median (range)	280 (67–950)	274 (142–950)	289 (67–837)	0.3
BMI ≥ 35, *n* (%)	9 (1.5%)	7 (1.5%)	2 (1.7%)	>0.9
Khorana risk, *n* (%)				0.001
Intermediate	418 (71%)	347 (74%)	71 (59%)	
High	171 (29%)	122 (26%)	49 (41%)	
Unknown	1	1	0	
Medical history of VTE/AMI/stroke, *n* (%)	11 (1.9%)	11 (2.3%)	0 (0%)	0.13
BMI > 30 kg/m^2^				
Mediastinal disease, *n* (%)	366 (62%)	269 (57%)	97 (81%)	<0.001
ECOG PS, *n* (%)				0.12
0–1	508 (86%)	410 (87%)	98 (82%)	
2–4	82 (14%)	60 (13%)	22 (18%)	
Extranodal disease, *n* (%)	126 (21%)	90 (19%)	36 (30%)	0.008
Leukopenia, *n* (%)	21 (3.6%)	20 (4.3%)	1 (0.8%)	0.095
Neutropenia, *n* (%)	3 (0.5%)	3 (0.6%)	0 (0%)	>0.9
Throly_Risk, *n* (%)				<0.001
Low‐risk	177 (30%)	161 (34%)	16 (13%)	
Int‐risk	344 (58%)	256 (55%)	88 (73%)	
High‐risk	69 (12%)	53 (11%)	16 (13%)	
Thrombotic event, *n* (%)	67 (11%)	57 (12%)	10 (8.3%)	0.2

Abbreviations: AMI, acute myocardial infarction; BMI, body mass index; ESR, erythrocyte sedimentation rate; GHSG, German Hodgkin Study Group; HTA, arterial hypertension; LDH, lactate dehydrogenase; PICC, peripherally implanted central venous catheter; PLTS, platelets; R/R, relapsing/refractory disease; VTE, venous thromboembolism.

^a^ Wilcoxon rank‐sum test, Pearson's chi‐square test, and Fisher's exact test.

One hundred twenty patients (20%) received primary prophylaxis with LMWH at diagnosis for clinical judgment. Patients who received prophylaxis were classified according to the Khorana score as follows: 59% (*n* = 71) at intermediate‐risk and 41% (*n* = 49) at high‐risk (Table 1). According to the Throly score, 13% (*n* = 16) were at low‐risk, 73% (*n* = 88) were at intermediate‐risk, and 13% (*n* = 16) were at high‐risk (Table 1). We observed 10/120 (8%) TE in patients receiving primary prophylaxis, 6 in intermediate‐risk patients, and 4 in high‐risk patients according to Khorana score (P > 0.9), all in intermediate‐risk patients according to ThroLy. Patients who received LMWH prophylaxis were excluded from further score validation analysis.

A total of 470 patients without prophylaxis were included in the analysis (Table 2 and Supporting Information S1: Figure 1). The median age at diagnosis of cHL was 37 years (range 17–85), with elderly (>60 years) accounting for 16% (*n* = 74) of the series. Eleven patients had a previous history of VTE/AMI/stroke (2%). Forty‐eight percent (*n* = 224) of patients had a PICC. For the nonprophylaxed subgroup, the median follow‐up was 3.4 (IQR 2.0–7.2).

**Table 2:** Characteristics of patients with no primary prophylaxis.

		Thrombotic event		
Characteristic	Overall *N* = 470	No *N* = 413	Yes *N* = 57	P value^a^
Age, median (range)	37 (17, 85)	36 (17, 85)	43 (17, 79)	0.058
Age, *n* (%)				0.4
<60	396 (84%)	350 (85%)	46 (81%)	
≥60	74 (16%)	63 (15%)	11 (19%)	
Age, *n* (%)				0.4
<65	420 (89%)	371 (90%)	49 (86%)	
≥65	50 (11%)	42 (10%)	8 (14%)	
Sex, *n* (%)				0.7
Male	228 (49%)	199 (48%)	29 (51%)	
Female	242 (51%)	214 (52%)	28 (49%)	
Ann Arbor stage, *n* (%)				0.3
I–II	251 (53%)	217 (53%)	34 (60%)	
III–IV	219 (47%)	196 (47%)	23 (40%)	
GHSG risk, *n* (%)				0.6
Early‐stage favorable	88 (19%)	77 (19%)	11 (19%)	
Early‐stage unfavorable	140 (31%)	119 (30%)	21 (37%)	
Advanced	225 (50%)	200 (51%)	25 (44%)	
B‐symptoms, *n* (%)	209 (44%)	183 (44%)	26 (46%)	0.9
Raised LDH, *n* (%)	126 (29%)	106 (27%)	20 (36%)	0.2
ESR, median (range)	44 (1, 140)	44 (1, 140)	40 (3, 120)	0.2
Unknown	31	23	8	
Bulky, *n* (%)	61 (13%)	45 (11%)	16 (28%)	<0.001
CVC/PICC employed, *n* (%)	224 (48%)	188 (46%)	36 (63%)	0.012
HTA under treatment, *n* (%)	67 (14%)	55 (13%)	12 (21%)	0.12
Diabetes under treatment, *n* (%)	23 (4.9%)	21 (5.1%)	2 (3.5%)	>0.9
R/R disease, *n* (%)	81 (17%)	64 (15%)	17 (30%)	0.007
Anemia, *n* (%)	73 (16%)	68 (16%)	5 (8.8%)	0.13
Leukocytosis, *n* (%)	177 (38%)	157 (38%)	20 (36%)	0.7
PLTS > 350, *n* (%)	175 (37%)	156 (38%)	19 (33%)	0.5
PLTs/mmc, median (range)	274,000 (142, 950,000)	280,000 (142, 950,000)	262,500 (183, 465,000)	0.6
BMI ≥ 35, *n* (%)	7 (1.5%)	4 (1.0%)	3 (5.3%)	0.042
Khorana risk, *n* (%)				0.6
Intermediate	347 (74%)	304 (74%)	43 (77%)	
High	122 (26%)	109 (26%)	13 (23%)	
Unknown	1	0	1	
Medical history of VTE/AMI/stroke, *n* (%)	11 (2.3%)	10 (2.4%)	1 (1.8%)	>0.9
BMI > 30, *n* (%)	34 (7.2%)	28 (6.8%)	6 (11%)	0.3
Mediastinal disease *n* (%)	269 (57%)	230 (56%)	39 (68%)	0.069
ECOG PS, *n* (%)				0.016
0–1	410 (87%)	365 (89%)	44 (77%)	
2–4	60 (13%)	47 (11%)	13 (23%)	
Extranodal disease, *n* (%)	90 (19%)	75 (18%)	15 (26%)	0.14
Leukopenia, *n* (%)	20 (4.3%)	18 (4.4%)	2 (3.6%)	>0.9
Neutropenia, *n* (%)	3 (0.6%)	3 (0.7%)	0 (0%)	>0.9
Throly_Risk, *n* (%)				0.4
Low‐risk	161 (34%)	146 (35%)	15 (26%)	
Int‐risk	256 (54%)	222 (54%)	34 (60%)	
High‐risk	53 (11%)	45 (11%)	8 (14%)	

Abbreviations: AMI, acute myocardial infarction; BMI, body mass index; CVC, cental venous catheter; ESR, erythrocyte sedimentation rate; GHSG, German Hodgkin Study Group; HTA, arterial hypertension; LDH, lactate dehydrogenase; PICC, peripherally implanted central venous catheter; PLTS, platelets; R/R, relapsing/refractory disease; VTE, venous thromboembolism.

^a^ Wilcoxon rank‐sum test, Fisher's exact test, and Pearson's chi‐square test.

Among the 470 patients, 57 (12%) experienced a TE, which was observed after a median follow‐up of 3.3 months (range 1–52) from cHL diagnosis: 21/470 were PICC‐related (4.5%) and 36/470 (7.66%) were not‐PICC‐related events. Among the latter, five were arterial thrombosis, and four PE.

TE was concomitant to diagnosis and staging in 23% (*n* = 13) of patients, and only 12% (*n* = 7) occurred after 10 months from diagnosis, concomitant with relapsing/refractory disease, confirming the robust association with active hematological disease (P < 0.001).

### Score validation

The cohort of 470 patients not receiving primary prophylaxis was stratified according to the Khorana and Throly risk scores (Table 3). According to Khorana criteria, no low‐risk patients were evidenced, intermediate‐risk were 74% (*n* = 347), high‐risk were 26% (*n* = 122); one was not classifiable for missing data. As for ThroLy criteria, low‐risk patients were 34% (*n* = 161), intermediate‐risk were 55% (*n* = 256), and high‐risk were 11% (*n* = 53).

**Table 3:** Throly and Khorana risk groups in patients with no primary prophylaxis.

		Thrombotic event		
Characteristic	Overall *N* = 470	No *N* = 413	Yes *N* = 57	P value^a^
Throly_Risk, *n* (%)				0.4
Low‐risk	161 (100%)	146 (91%)	15 (9.3%)	
Int‐risk	256 (100%)	222 (87%)	34 (13%)	
High‐risk	53 (100%)	45 (85%)	8 (15%)	
Khorana risk, *n* (%)				0.6
Intermediate	347 (100%)	304 (88%)	43 (12%)	
High	122 (100%)	109 (89%)	13 (11%)	
Unknown	1	0	1	

^a^ Pearson's chi‐square test.

No correlation was found between Khorana categories and the occurrence of TE. High‐risk patients showed a comparable rate of TE compared to intermediate, 11% (13/122) versus 12% (43/347), respectively (P = 0.6) (Table 3). Same results were observed stratifying patients according to ThroLy: TE occurred in 15% (8/53) of high‐risk patients, versus 13% (34/256) and 9% (15/161) in intermediate‐ and low‐risk cases, respectively (P = 0.4).

### Thrombotic risk factors

Thus, we performed univariate analysis to assess the value of single disease and patients' features that could influence the thrombotic risk.

First, each single risk factor included in the ThroLy score was analyzed. Mediastinal involvement had a trend for an increased risk of TE (bulky or not bulky) with OR 1.72 (CI 0.97–3.18, P = 0.07). Patients with ECOG PS between 2 and 4 presented the highest rate of events, with an OR of 2.29 (CI 1.12–4.48, P = 0.02) (Tables 2, 3, 4). Conversely, anemia (P = 0.14), neutropenia (P > 0.9), BMI > 30 (P = 0.31), and previous history of VTE/AMI/Stroke (P = 0.76) were not confirmed as prognostic factors in univariate analysis.

**Table 4:** Univariate and multivariate analyses in patients with no primary prophylaxis, according to the occurrence of a thrombotic event.

	Univariate					Multivariate		
Characteristic	*N*	Event *N*	OR^a^	95% CI^a^	P value	OR^a^	95% CI^a^	P value
Age	470	57	1.01	1.00, 1.03	0.094	1.02	1.00, 1.04	0.059
Age	470	57						
<60			—	—				
≥60			1.33	0.62, 2.62	0.43			
Age	470	57						
<65			—	—				
≥65			1.44	0.60, 3.11	0.38			
BMI > 30	470	57	1.62	0.58, 3.85	0.31			
Medical history VTE/AMI/stroke	470	57	0.72	0.04, 3.86	0.76			
Bulky	470	57	3.19	1.62, 6.07	<0.001	2.98	1.46, 5.90	0.002
Mediastinal disease	470	57	1.72	0.97, 3.18	0.071	1.84	0.97, 3.57	0.065
Extranodal disease	470	57	1.61	0.83, 3.00	0.15			
ECOG PS	469	57						
0–1			—	—		—	—	
2–4			2.29	1.12, 4.48	0.018	2.19	0.99, 4.65	0.047
Cohort	470	57						
1			—	—				
2			0.84	0.48, 1.49	0.55			
Sex	470	57						
Male			—	—				
Female			0.90	0.51, 1.57	0.70			
Ann Arbor stage	470	57						
I–II			—	—				
III–IV			0.75	0.42, 1.31	0.31			
B‐symptoms	470	57	1.05	0.60, 1.84	0.85			
Leukocytosis	469	56	0.91	0.50, 1.61	0.74			
Leukopenia	469	56	0.81	0.13, 2.92	0.78			
Anemia	470	57	0.49	0.17, 1.16	0.14			
PLTS > 350 × 10^9^/L	470	57	0.82	0.45, 1.46	0.52			
HTA under treatment	470	57	1.74	0.83, 3.40	0.12			
Diabetes under treatment	470	57	0.68	0.11, 2.40	0.61			
PICC employed	470	57	2.05	1.17, 3.69	0.014	2.21	1.23, 4.09	0.009
R/R disease	470	57	2.32	1.21, 4.28	0.009			

Abbreviations: AMI, acute myocardial infarction; BMI, body mass index; HTA, arterial hypertension; PICC, peripherally implanted central venous catheter; PLTS, platelets; R/R, relapsing/refractory disease; VTE, venous thromboembolism.

^a^ OR = odds ratio; CI = confidence interval.

Moreover, no significant association with TE was found related to sex (P = 0.70), stage (P = 0.31), histology subtype (P = 0.71), leucocytosis (P = 0.74), diabetes under treatment (P = 0.61), B‐symptoms (P = 0.8), and arterial hypertension (HTA) under treatment (P = 0.12). A significant association was found related to bulky disease and presence of PICC: bulky disease, mediastinal or not, was associated with an OR of 3.19 (CI 1.62–6.07, P < 0.001), whereas PICC employment was associated with an increased OR of 2.05 (CI 1.17–3.69, P = 0.014) (Tables 2, 3, 4).

Age as a continuous variable showed a trend of association with TE (P = 0.09). A subanalysis was also performed according to elderly patients' subgroups, aged > 60 years (P = 0.43) and >65 years (P = 0.38), with no significant difference in terms of TE rate (Tables 2, 3, 4).

In addition, continuous variables at diagnosis, such as Hb, white blood cell count, platelets, lactate dehydrogenase, and erythrocyte sedimentation rate (ESR), were compared in relation to the thrombotic risk, but no significant association emerged (Tables 2, 3, 4).

### Multivariate analysis and Thro‐HL score

We performed a multivariate analysis including all the single risk factors that resulted in significant or with trend (P < 0.09) at univariate analysis: age, bulky disease (defined as a nodal mass with more than 7 cm of diameter, regardless of the anatomical site), mediastinal involvement (regardless of size), PICC employment, and high ECOG PS. Multivariate regression confirmed the independent prognostic impact of each factor, except for age and the borderline significance of mediastinal involvement (P = 0.065) (Table 4).

Considering the OR of the multivariate analysis and selecting the most significant, we then outlined a new risk model (called Thro‐HL) including the following four risk factors: bulky disease (3 points), high ECOG PS (2 points), presence of PICC (2 points), and mediastinal involvement (1 point). This model had an AUC of 0.74 (bootstrap 95% CI 0.68–0.79): low‐risk was defined by a score of 0–1, intermediate‐risk was defined by a score of 2–3, and high‐risk as a score > 3.

According to this new stratification, performed on 469/470 patients with complete information, 39% (*n* = 183) were low‐risk, 46% (*n* = 214) were intermediate‐risk, and 15% (*n* = 72) were high‐risk. The rate of TE was significantly different between categories, 2.7% in low‐risk (5/183), 16% in intermediate‐risk (34/214), and 25% in high‐risk (18/72) (P < 0.001). Kaplan–Meier curve in Figure 1 shows differences in terms of 3 year‐t‐EFS ranging from 97% (CI 95–100) for low‐risk to 76% (CI 66–86) for high‐risk (P < 0.0001). The score was tested for Harrel's *C*‐index, which resulted in 0.70 (SD = 0.03).

A subanalysis was performed in the subgroup of 74 (15.8%) patients aged ≥ 60 years with no significant difference in terms of t‐EFS (P = 0.15). Among elderly patients, mediastinal involvement was less frequent (P < 0.001) and ECOG PS was higher (P < 0.001) (Table 5).

**Table 5:** Distribution of Thro‐HL clinical features in elderly patients.

		Age		
Characteristic	Overall *N* = 470	<60 *N* = 396	≥60 *N* = 74	P value^a^
Score, *n* (%)				0.4
Low	183 (39%)	159 (40%)	24 (32%)	
Int	214 (46%)	177 (45%)	37 (50%)	
High	72 (15%)	59 (15%)	13 (18%)	
Unknown	1	1	0	
Thrombotic event, *n* (%)	57 (12%)	46 (12%)	11 (15%)	0.4
Bulky, *n* (%)	61 (13%)	54 (14%)	7 (9.5%)	0.3
ECOG PS, *n* (%)				<0.001
0–1	409 (87%)	362 (92%)	47 (64%)	
2–4	60 (13%)	33 (8.4%)	27 (36%)	
Unknown	1	1	0	
Mediastinal disease, *n* (%)	269 (57%)	243 (61%)	26 (35%)	<0.001
PICC employed, *n* (%)	224 (48%)	194 (49%)	30 (41%)	0.2

Abbreviation: PICC, peripherally implanted central venous catheter.

^a^ Pearson's chi‐square test.

Since PICC‐related thrombosis (21 events) can be considered associated with different risk factors and etiopathogenesis than the other VTE, we also tested the performance of our Thro‐HL score in predicting the non‐PICC‐related TE (36 events). The rate of TE was significantly different between categories, 2.7% in low‐risk (5/183), 9.5% in intermediate‐risk (19/199), and 18% in high‐risk (12/66) (P < 0.001) (Supporting Information S3: Table 1). Three‐year t‐EFS was 97% (CI 95–100) for low‐risk and 83% (CI 74–93) for high‐risk (P = 0.0002) (Supporting Information S2: Figure 2).

In the subgroup of 120 patients receiving LMWH and excluded from the analysis, 81 (68%) were classified as high‐risk according to Thro‐HL; primary prophylaxis with LMWH abrogated the thrombotic risk (P = 0.7).

## DISCUSSION

Thrombosis in lymphomas increases morbidity and mortality, but nonspecific administration of thromboprophylaxis can increase the risk of bleeding complications. There is a need for a predictive risk model specific for cHL, on which indication for thromboprophylaxis can be based.

The thrombotic rate reported in patients with cHL ranges from 3.3% to 10%.^11^, ^13^, ^14^, ^18^, ^22^, ^23^, ^24^ Few analyses have been conducted on the thrombotic risk in cohorts including exclusively cHL patients.^13^, ^24^

In our study, focused on 470 patients with cHL and not receiving primary prophylaxis with LMWH, we report an overall TE incidence of 12%. TE occurred early during front‐line therapy (median time 3.3 months), confirming that the thrombotic risk is an early event firmly linked to an active disease.^13^, ^23^ Regarding chemotherapy, most patients received the ABVD/AVD regimen; thus, the impact of dose‐dense regimens could not emerge.^13^

We first applied Khorana and ThroLy scores to evaluate their ability to assess the thrombotic risk in cHL. Applying the Khorana score to our cohort, no patient was low‐risk due to the diagnosis of lymphoma in itself. Thus, the majority of patients were included in the intermediate‐risk (74%), with the remaining 26% in the high‐risk category. We did not observe a significant difference in the thrombotic rate between the two groups (11% high‐risk vs. 12% intermediate‐risk). Similar results were reported from other experiences, which included diffuse large B‐cell lymphomas (DLBCLs) and cHL, in which the incidence of TE was not statistically different between high‐risk and intermediate‐risk patients according to Khorana score (17% vs. 15%)^18^ and even in series including only cHL patients, where Khorana was not predictive.^13^, ^24^ Khorana score, despite its applicability in clinical practice, results are insufficient for a clinically relevant stratification of cHL, given that the large group of intermediate‐risk patients could be better stratified.^13^, ^21^, ^24^

Applying to our series the Throly score, which was conceived for lymphoma patients, low‐risk patients were 34%, intermediate‐risk patients were 55%, and high‐risk patients were 11%. No difference in the TE rate was observed, since TE occurred in 9% of 161 low‐risk cases, 13% of 256 intermediate, and in 15% of 53 high‐risk patients, respectively (P = 0.4).

Rupa‐Matysek et al.^19^ retrospectively assessed the ThroLy score in DLBCL and HL: intermediate‐risk patients showed a 29% thrombotic rate, in contrast to 13% found in our cohort. The authors did not confirm the ThroLy score's predictive significance on the basis of the poor discriminating power between intermediate and high‐risk patients. However, pooling together two subgroups of lymphoma can expose to some biases. cHL patients are usually younger and with fewer comorbidities than NHL patients, with a lower incidence of individual patient‐related factors for VTE (e.g., obesity, heart or renal failure, and poor performance status).^9^ Moreover, bone marrow involvement is less frequent in cHL than NHL; consequently, cytopenias, identified as a potential risk factor by the Throly score, are rare. In addition, a higher thrombotic risk in DLBCL than in cHL patients has been reported.^11^, ^14^, ^18^, ^19^, ^20^

Ma'koseh et al.^24^ classified 321 cHL patients in Throly low‐risk (9.3%), intermediate (62%), and high‐risk (29%), with no correlation between high‐risk and VTE, confirming our results.

We performed univariate and multivariate analyses to investigate other potential parameters that could be included in cHL‐specific risk stratification.

Taking into account one by one the single risk factors included in the ThroLy score (i.e., previous VTE/AMI/stroke, reduced mobility [ECOG 2–4], obesity, extranodal localization, mediastinal involvement, neutrophils < 1 × 10^9^/L, and hemoglobin level < 100 g/L),^14^ in our series only ECOG PS and mediastinal involvement were prognostic factors of TE.

We also evaluated the impact of PICC on TE, as many studies reported an association between VTE and PICC, especially in patients with lymphoma.^25^ In our series, 63% of patients with thrombosis had a PICC. The rate of PICC‐related TE was 4.5%. Indeed, this variable retained an independent value in multivariate analysis.

Other thrombotic risk factors, such as HTA on treatment or diabetes, were not associated with a significant thrombotic rate. Neither the available inflammation‐related parameters, such as ESR or platelet number, predicted TE.

We identified four independent risk factors: bulky disease (3 points), high ECOG PS (2 points), presence of PICC (2 points), and mediastinal involvement (1 point). Weighting the HR of each of these factors, we included these four characteristics in a disease‐specific Thro‐HL model, which is promising in distinguishing real low‐risk (39%) from intermediate (46%) and high‐risk (15%) patients, with a significantly different rate of TE, of 2.7%, 16%, and 25%, respectively. The significance of Thro‐HL score held also after exclusion of PICC‐related TE.

Interestingly, ECOG ≥ 2 and bulky disease were also independently associated with VTE in a series of 857 patients with both NHL and HL requiring hospitalization.^26^

In clinical practice, LMWH prophylaxis is a clinical decision made by the physician usually in lymphoma patients with bulky disease or venous compression. However, no guidelines on specific thromboprophylaxis measures are available in lymphomas as well as in hematologic neoplasms in general.^27^ Although primary thrombosis prophylaxis is mandatory for direct cancer‐related vessel compression, its employment in patients with large masses is not.^28^, ^29^ The ASH guidelines suggest parenteral thromboprophylaxis with LMWH for outpatients with cancer at high risk of TE receiving systemic therapy.^29^ In Italian clinical practice, LMWH prophylaxis is based on the Khorana score, as recommendation and refundability for LMWH prescription is allowed only for patients with a high risk of TE (Khorana score > 3).^30^

Our study well reflects a degree of heterogeneity in the application of thromboprophylaxis in patients with cHL. LMWH primary thromboprophylaxis was performed in 20% of patients with cHL; these patients were excluded from the analysis to avoid a possible bias, similar to other series^14^, ^18^, ^20^ and in contrast with other studies in which these data were lacking.^13^, ^26^ The clinical judgment concerning primary prophylactic LMWH administration was heterogeneous, since 61 patients (13%) with bulky disease in our cohort received no LMWH administration. Since bulky disease, independently from the anatomical localization and direct vessel compression, is an independent prognostic factor for thrombotic risk, this would support the employment of thromboprophylaxis in this subset of cHL patients. On the other hand, a predictive score capable of identifying a low‐risk category of patients with a TE rate less than 3% can be useful to spare an unnecessary thromboprophylaxis in a not negligible proportion of cHL patients.

In conclusion, our findings show that Khorana and ThroLy scores are not able to predict the thrombotic risk in cHL patients treated with ABVD/AVD regimens outside clinical trials. Our Thro‐HL model includes four patients' related parameters, which are easily assessable by the physician in the daily routine, making this score highly feasible and disease‐specific. Our study has some limitations, given the retrospective nature and the lack of a validation cohort. Thro‐HL could be a promising tool that should be validated in large prospective cohorts to determine which patients would benefit the most from primary prophylaxis from diagnosis (which appears to be the time with the highest risk of TE, according to our findings) and which could avoid it. Moreover, prospective studies are also needed to determine if LMWH prophylaxis is required for the entire duration of the front‐line treatment or until evidence of response according to the type of treatment scheme and in disease‐specific groups.

## AUTHOR CONTRIBUTIONS

**Giovanni Manfredi Assanto**: Conceptualization; investigation; writing—original draft; methodology; writing—review and editing; data curation; supervision; visualization. **Eleonora Alma**: Investigation; data curation; resources; writing—review and editing. **Alessandro Cellini**: Investigation; writing—review and editing; data curation; resources. **Giovanni Marsili**: Methodology; validation; software; formal analysis; project administration; data curation; investigation. **Gianluca Maiorana**: Data curation; resources; investigation. **Cristina Santoro**: Investigation; conceptualization; formal analysis; data curation. **Martina Salvatori**: Investigation; writing—review and editing; data curation. **Natalia Cenfra**: Investigation; data curation; writing—review and editing. **Vladimir Otasevic**: Investigation; writing—review and editing; data curation. **Darko Antic**: Investigation; writing—review and editing; data curation; resources. **Gianna Maria D'Elia**: Investigation; writing—review and editing; data curation. **Maria Paola Bianchi**: Investigation; writing—review and editing; data curation. **Giorgia Annechini**: Investigation; data curation. **Silvio Ligia**: Investigation; data curation. **Alessandro Pulsoni**: Investigation; writing—review and editing; supervision; data curation. **Agostino Tafuri**: Investigation; writing—review and editing; visualization; supervision. **Andrea Visentin**: Investigation; writing—review and editing; data curation; supervision. **Alfonso Piciocchi**: Investigation; methodology; validation; visualization; software; formal analysis; supervision. **Stefan Hohaus**: Investigation; methodology; writing—original draft; writing—review and editing; supervision. **Maurizio Martelli**: Investigation; writing—review and editing; supervision. **Ilaria Del Giudice**: Writing—original draft; writing—review and editing; conceptualization; investigation; supervision; data curation; resources; project administration; visualization; methodology. **Antonio Chistolini**: Investigation; conceptualization; writing—review and editing; supervision; data curation.

## CONFLICT OF INTEREST STATEMENT

The authors declare no conflicting financial interest with the submission of this article. G.M.A., G.M.D., A.Pu., A.V., and I.D.G. attended a scientific board organized by Takeda.

## ETHICS STATEMENT

This study was approved by the local ethical committee and respects the principles of the Declaration of Helsinki.

## FUNDING

The authors declare no sources of funding to perform this retrospective study, which is not a clinical trial.

## Supporting information

Supporting Information.

Supporting Information.

Supporting Information.

Supporting Information.

## Data Availability

The data that support the findings of this study are available on request from the corresponding author. The data are not publicly available due to privacy or ethical restrictions.
