# Supplementary figures and images for: Predicting thrombotic risk in patients with classical Hodgkin lymphoma: Thro‐HL multicenter study
Source: Hemasphere. 2025 Jul 13;9(7):e70163. doi: 10.1002/hem3.70163 (PMC12255904; doi:10.1002/hem3.70163)

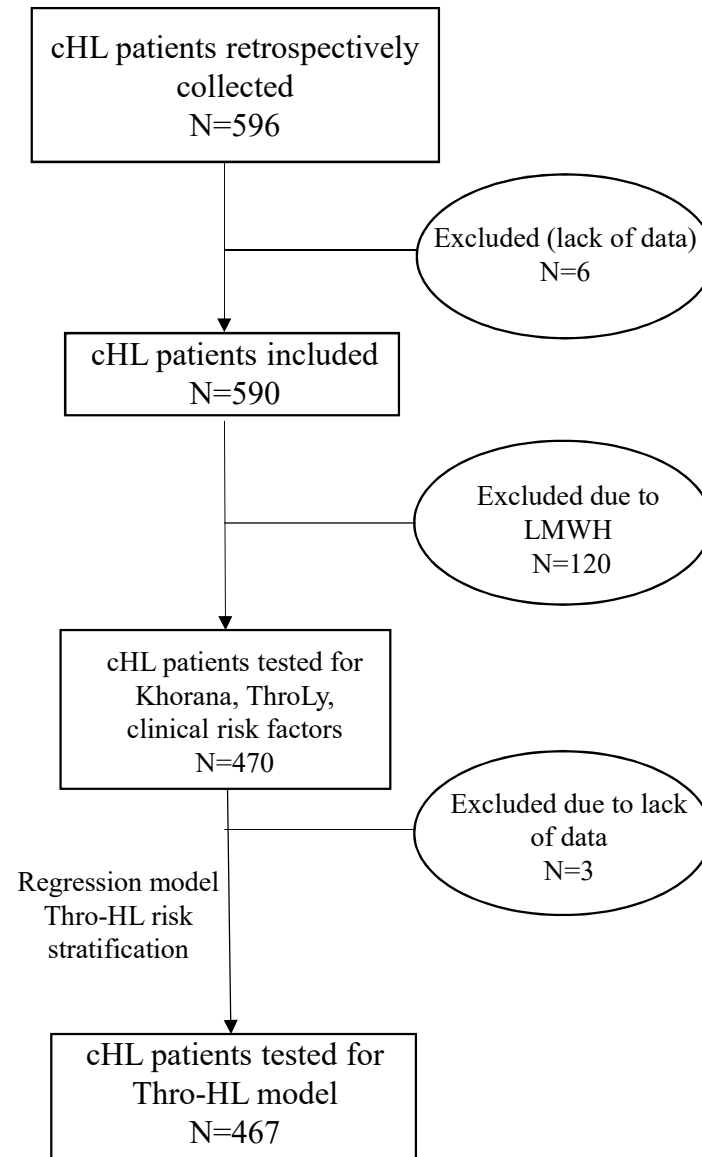

Supplement: Supplementary file 1 — Supporting Information. [file HEM3-9-e70163-s004.pdf]
