# Supplementary material for: Predicting thrombotic risk in patients with classical Hodgkin lymphoma: Thro‐HL multicenter study
Source: Hemasphere. 2025 Jul 13;9(7):e70163. doi: 10.1002/hem3.70163 (PMC12255904; doi:10.1002/hem3.70163)
Supplement: Supplementary file 2 — Supporting Information. [file HEM3-9-e70163-s003.docx]

## Supplementary Figure 2. Event Free Survival of patients with no primary prophylaxis according to the Thro-HL score excluding PICC-related thrombotic event (TE). Event is considered a non-PICC related TE.


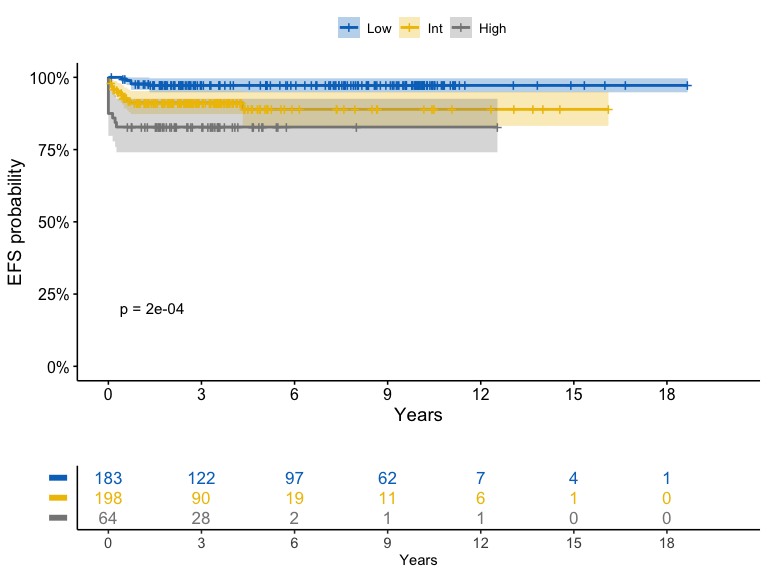


EFS Cox Regression.

| **Characteristic** | **3 years** | **HR**^1^ | **95% CI**^1^ | **p-value** |
| --- | --- | --- | --- | --- |
| **Score** |  |  |  |  |
| *Low* | 97% (95%, 100%) | — | — |  |
| *Int* | 91% (87%, 95%) | 3.68 | 1.36, 9.94 | **0.010** |
| *High* | 83% (74%, 93%) | 7.54 | 2.61, 21.8 | **<0.001** |
|  |  |  |  |  |
