# Supplementary material for: Predicting thrombotic risk in patients with classical Hodgkin lymphoma: Thro‐HL multicenter study
Source: Hemasphere. 2025 Jul 13;9(7):e70163. doi: 10.1002/hem3.70163 (PMC12255904; doi:10.1002/hem3.70163)
Supplement: Supplementary file 3 — Supporting Information. [file HEM3-9-e70163-s002.docx]

# **Supplementary Table 1.** Thro-HL score in patients with no primary prophylaxis, according to the occurrence of a non-PICC related Thrombotic Event.

|  | | **Non-PICC related**  **Thrombotic event** | |  |
| --- | --- | --- | --- | --- |
| **Characteristic** | **Overall**  N = 449 | **No** N = 413 | **Yes** N = 36 | **p-value**^1^ |
| **Score, n (%)** |  |  |  | **<0.001** |
| *Low* | 183 (100%) | 178 (97%) | 5 (2.7%) |  |
| *Int* | 199 (100%) | 180 (90%) | 19 (9.5%) |  |
| *High* | 66 (100%) | 54 (82%) | 12 (18%) |  |
| *Unknown* | 1 | 1 | 0 |  |
| ^1^Pearson's Chi-squared test; Fisher's exact test | | | | |
