# Supplementary material for: Predicting thrombotic risk in patients with classical Hodgkin lymphoma: Thro‐HL multicenter study
Source: Hemasphere. 2025 Jul 13;9(7):e70163. doi: 10.1002/hem3.70163 (PMC12255904; doi:10.1002/hem3.70163)
Supplement: Supplementary file 4 — Supporting Information. [file HEM3-9-e70163-s001.docx]

## Legend Supplementary Figure 1. Consort diagram of enrolled population of classical Hodgkin Lymphoma (cHL) patients. From a starting population of 596 consecutive patients, six were excluded due to lack of data, 120 were excluded from risk analysis due to low molecular weight heparin (LMWH) prophylaxis to exclude possible bias. Thrombosis Hodgkin Lymphoma (Thro-HL), Khorana and ThroLy scores were tested on 467 patients.
